# ATP-Induced Inflammasome Activation and Pyroptosis Is Regulated by AMP-Activated Protein Kinase in Macrophages

**DOI:** 10.3389/fimmu.2016.00597

**Published:** 2016-12-12

**Authors:** Qing-Bing Zha, Hong-Xia Wei, Chen-Guang Li, Yi-Dan Liang, Li-Hui Xu, Wen-Jing Bai, Hao Pan, Xian-Hui He, Dong-Yun Ouyang

**Affiliations:** ^1^Department of Fetal Medicine, The First Affiliated Hospital of Jinan University, Guangzhou, China; ^2^Department of Immunobiology, College of Life Science and Technology, Jinan University, Guangzhou, China; ^3^Department of Cell Biology, College of Life Science and Technology, Jinan University, Guangzhou, China

**Keywords:** AMP-activated protein kinase, bacterial sepsis, inflammasome, metformin, pyroptosis

## Abstract

Adenosine triphosphate (ATP) is released by bacteria and host cells during bacterial infection as well as sterile tissue injury, acting as an inducer of inflammasome activation. Previous studies have shown that ATP treatment leads to AMP-activated protein kinase (AMPK) activation. However, it is unclear whether AMPK signaling has been involved in the regulation of ATP-induced inflammasome activation and subsequent pyroptosis. In this study, we aimed to investigate this issue in lipopolysaccharide-activated murine macrophages. Our results showed that AMPK signaling was activated in murine macrophages upon ATP treatment, which was accompanied by inflammasome activation and pyroptosis as evidenced by rapid cell membrane rupture as well as mature interleukin (IL)-1β and active caspase-1p10 release. The ATP-induced inflammasome activation and pyroptosis were markedly suppressed by an AMPK inhibitor compound C or small-interfering RNA-mediated knockdown of *AMPK*α, but could be greatly enhanced by metformin (a well-known AMPK agonist). Importantly, metformin administration increased the mortality of mice with bacterial sepsis, which was likely because metformin treatment enhanced the systemic inflammasome activation as indicated by elevated serum and hepatic IL-1β levels. Collectively, these data indicated that the AMPK signaling positively regulated ATP-induced inflammasome activation and pyroptosis in macrophages, highlighting the possibility of AMPK-targeting therapies for inflammatory diseases involving inflammasome activation.

## Introduction

Inflammasomes are large multimeric protein complexes present in the cytosol of immune cells to sense and respond to pathogen infection or tissue injury. Their activation constitutes a first line of defense against microbial infection (1). One of the most extensively investigated inflammasomes is nucleotide and oligomerization domain, leucine-rich repeat containing protein family, pyrin containing domain 3 (NLRP3) in innate immune cells including macrophages. The full activation of NLRP3 inflammasome requires two steps. The first step is to prime the macrophages with pathogen-associated molecular patterns (PAMPs) which are recognized by specific pattern recognition receptors (PRRs) (2). For example, lipopolysaccharide (LPS), a well-known PAMP expressed on Gram-negative bacteria, binds to and activates toll-like receptor 4 leading to the upregulation of critical inflammasome components (e.g., NLRP3 protein) (3). The second step is to trigger these primed cells by danger signals, such as adenosine triphosphate (ATP) and uric acid crystal, culminating in the assembly of NLRP3 inflammasome (1). This is the canonical pathway of NLRP3 inflammasome activation. Recently, it has been demonstrated that LPS can directly bind to and activate caspase-11 when LPS is released into the cytosol from engulfed bacteria *via* as-yet-unrecognized routes or by artificial transfection into the cytosol (4), which subsequently leads to the non-classical NLRP3 inflammasome activation (3, 5).

Following the assembly of inflammasomes, intracellular caspase-1 is activated to catalyze pro-interleukin-1β (pro-IL-1β) cleavage into mature IL-1β (1). As a canonical activator of NLRP3 inflammasome, ATP can be released from both host and bacterial cells in the circumstance of bacterial infection (6) or tissue injury (7, 8). Upon PAMP stimulation, monocytes/macrophages can release endogenous ATP into the extracellular milieu (8), and they can also produce carbon monoxide (CO) to promote bacterial ATP release, resulting in the activation of NLRP3 inflammasome and maturation of IL-1β to intensify bacterial killing (9). Notably, the release of mature IL-1β is reliant on pyroptosis (10). Pyroptosis is another consequence of caspase-1 activation, which is characterized by rapid cell swelling and membrane rapture, leading to the release of intracellular contents (11). Therefore, inflammasome activation represents robust inflammatory responses during bacterial infection or tissue injury, which should benefit the host to clear the pathogens or repair the injured tissues by recruiting various inflammatory immune cells. However, sustained inflammasome activation by endogenous danger signals released from damaged cells may aggravate the pathological inflammation in sterile inflammatory disorders (12–14). It has been demonstrated that inflammasome activation and pyroptosis can also take place in multiple organs after systemic infection (sepsis) (15, 16), implicating pyroptosis as one cause of multiple organ failure in septic patients (3).

Although NLRP3 inflammasome activation has a critical role in bacterial infection or other pathological conditions, the signaling pathways regulating this process are still elusive. The fact that septic patients usually require nutrition supplement in clinic suggests that energy metabolism may influence cell survival in sepsis (17). Among the regulators of cellular energy metabolism, AMP-activated protein kinase (AMPK) is a key nexus highly conserved in eukaryotic cells for sensing and responding to cellular energy status (18). It is a heterotrimeric protein comprised of α, β, and γ subunits, and the α subunit contains the catalytic domain, in which phosphorylation at threonine (Thr) 172 makes this kinase activated (19). The activation of AMPK takes place when the cellular AMP/ATP ratio is elevated due to metabolic stresses (e.g., insufficient glucose and oxygen supply) or ATP consumption (e.g., muscle contraction). It regulates not only cellular metabolism but also cell survival and proliferation (20). Therefore, we hypothesized that AMPK might have been involved in the regulation of ATP-induced inflammasome activation and pyroptosis. Indeed, recent studies have indicated that bacterial infection or ATP treatment in LPS-primed macrophages can dramatically elevate AMPK activation, although LPS *per se* suppresses the activity of AMPK (21–23).

In this study, we aimed to investigate whether AMPK activation contributed to ATP-induced inflammasome activation in LPS-primed murine macrophages. Our results demonstrated that enhancing AMPK activation by metformin (an agonist of AMPK) sensitized LPS-primed macrophages to pyroptosis upon ATP treatment, while suppression of AMPK activity by its pharmacologic inhibitor compound C or *AMPK*α-specific small-interfering RNA (siRNA) reduced the ratio of pyroptosis. Moreover, metformin administration to mice significantly increased the systemic levels of IL-1β and animal mortality upon bacterial infection. Our data indicated that AMPK signaling had been involved in ATP-induced inflammasome activation and pyroptosis in LPS-primed macrophages.

## Materials and Methods

### Reagents and Animals

DMEM, Opti-MEM, l-glutamine, streptomycin, penicillin, and fetal bovine serum (FBS) were obtained from Thermo/Fisher/Gibco (Carlsbad, CA, USA). LPS (*Escherichia coli* O111:B4) (L4391), ATP (P8232), dimethyl sulfoxide, Hoechst 33342, propidium iodide (PI), Tween-80, and compound C were bought from Sigma-Aldrich (St. Louis, MO, USA). Metformin was obtained from MedChem Express (Princeton, NJ, USA), dissolved in PBS at 300 mM and stored at −20°C. The working solution of metformin was freshly prepared. Antibodies against IL-1β (#12242), HMGB1 (#3935), AMPKα (#5832), phospho(p)-AMPK (Thr172) (#2535), β-tubulin (#2128), ASC (#67824), and horseradish peroxidase-conjugated goat anti-mouse/rabbit IgG were bought from Cell Signaling Technology (Danvers, MA, USA). The antibody to NLRP3 (AG-20B-0014) was purchased from Adipogen AG (Liestal, Switzerland). The antibody against caspase-1p10 (sc-514) was obtained from Santa Cruz (Santa Cruz Biotechnology, Dallas, TX, USA). Anti-mouse CD11b FITC (11-0112) and anti-mouse Ly-6G (Gr-1) PE (12-9668) were obtained from eBioscience (San Diego, CA, USA). C57BL/6 mice (6–8 weeks of age) were purchased from the Laboratory Animal Center of Southern Medical University (Guangzhou, China). All animal experiments were performed according to the guidelines for the care and use of animals approved by the Committee on the Ethics of Animal Experiments of Jinan University.

### Cell Line Culture

Mouse macrophage cell line J774A.1 was obtained from the Kunming Cell Bank of Type Culture Collection Chinese Academy of Sciences (Kunming, China). The cells were maintained in complete DMEM medium (containing 10% FBS, 100 µg/ml penicillin, 100 µg/ml streptomycin, and 2 mM l-glutamine) and cultured at 37°C in a humidified incubator with 5% CO_2_. The cells were subcultured every 2–3 days.

### Bone Marrow-Derived Macrophages (BMDMs)

Bone marrow-derived macrophages from C57BL/6 mice were differentiated as reported previously (3). Briefly, mouse bone marrow cells were differentiated in DMEM supplemented with 10% FBS and 20% M-CSF-conditioned medium from L929 cells for 6 days. The BMDMs were then cultured overnight in 24-well plates at 1.8 × 10^5^ cells/well in 0.5 ml medium.

### Determination of Soluble Cytokines

Soluble IL-1β in cell culture medium and orbital serum were determined by cytometric bead array (CBA) mouse IL-1β flex set (#560232) according to the manufacturer’s instructions (BD Biosciences, San Jose, CA, USA).

### Phenotype Analysis

Cells were washed twice with PBS-F (PBS containing 0.1% NaN_3_ and 3% FBS), then stained with FITC-labeled anti-CD11b and PE-labeled anti-mouse Ly-6G monoclonal antibodies for 30 min. Red blood cells, if there were, were lysed. After washing with PBS-F, the cells were fixed with 4% paraformaldehyde in PBS and then analyzed by a flow cytometer (FACSCalibur; Becton Dickinson).

### Pyroptosis Assay

Cell pyroptosis was measured as described previously (24, 25). Briefly, cells were seeded in 24-well plates and cultured for 24 h. The cells were then primed with 500 ng/ml LPS for 4 h followed by treatment with indicated concentrations of metformin in Opti-MEM for 1 h. Next, indicated concentrations of ATP were added to the culture medium for indicated time periods. Subsequently, the cells were stained with staining solution containing PI (2 µg/ml) and Hoechst 33342 (5 µg/ml). Dead cells (PI permeable) were observed under a Zeiss Axio Observer D1 microscope and fluorescence images were captured by the Zeiss AxioCam MR R3 cooled CCD camera controlled with ZEN software (ZEISS, Germany).

### Precipitation of Soluble Proteins

Precipitation of soluble proteins was performed as previously described (26). In brief, proteins existing in cell culture supernatants were precipitated overnight with 7.2% trichloroacetic acid and 0.15% sodium deoxycholate solutions. After washing three times with cold acetone, the precipitated proteins were redissolved in sodium dodecyl sulfate-polyacrylamide gel electrophoresis (SDS-PAGE) loading buffer and subjected to western blot analysis.

### Western Blot Analysis

Whole cell lysates and western blotting were performed as previously described (27). Cells were lysed with 2× SDS-PAGE loading buffer, and the total proteins were separated by SDS-PAGE and then transferred onto polyvinylidene difluoride membranes (Hybond-P; GE Healthcare Life Sciences, Piscataway, NJ, USA). The membrane was blocked and incubated with primary antibody overnight, followed by incubation with horseradish peroxidase-conjugated goat anti-rabbit IgG or goat anti-mouse IgG. Bands on the membrane were revealed by a BeyoECL Plus kit (Beyotime, P0018) and recorded on X-ray films (Kodak, 6535873). Images were captured by using FluorChem 8000 Imaging System (AlphaInnotech, San Leandro, CA, USA), and densitometry of each band was quantified using AlphaEaseFC software (AlphaInnotech).

### Immunofluorescence Microscopy

Bone marrow-derived macrophages were planted in glass-bottomed dishes (5 × 10^5^ cells/dish) and cultured at 37°C overnight. Then the cells were primed with 500 ng/ml LPS for 4 h, followed by treatment with 2 mM metformin or vehicle (PBS) for 1 h. Subsequently, 2 mM ATP was added to the culture medium for 30 min. After these treatments, the cells were fixed in 4% paraformaldehyde for 15 min and permeabilized with 2 ml cold methanol (−20°C) for 10 min. Then the cells were incubated with ASC antibody (1:300) overnight, followed by staining with CF568-conjugated goat-anti-rabbit IgG (#20103) (Biotium, Hayward, CA, USA) for 1 h. Finally, Hoechst 33342 solution (5 µg/ml in PBS) was added to stain the nuclei for 10 min. The cells were observed under a Zeiss Axio Observer D1 microscope with a Zeiss LD Plan-Neofluar 40×/0.6 Korr M27 objective (Carl Zeiss MicroImaging GmbH, Göttingen, Germany). Fluorescence images were captured with a Zeiss AxioCam MR R3 cooled CCD camera controlled with ZEN software (ZEISS).

### Knockdown of AMPKα

The siRNA (5′-GCA GAA GUG UGU AGA GCA A-3′) duplexes targeting mouse *AMPK*α*1* and negative control (NC) siRNA were designed and synthesized by RiboBio (Guangzhou, China). Knockdown of AMPKα1 was performed according to the instructions provided by the manufacturers. Briefly, transfection reagent Lipofectamine RNAiMAX (Invitrogen, 13778-075) and AMPKα1 siRNA were diluted in Opti-MEM, respectively, and then mixed together. The NC siRNA mixture was also prepared. Then the cell culture medium was replaced with Opti-MEM, and the siRNA mixture was added at a final siRNA concentration of 100 nM. Six hours later, the cells were cultured in normal medium containing 10% FBS. After being cultured for additional 72 h, the cells were ready for experiments.

### Bacterial Infection

Bacterial sepsis mouse model was established as previously described (9, 27). Briefly, C57BL/6 mice were acclimated for 1 week. Bacteria (*E. coli* DH5α) were cultured and proliferated in Luria Broth medium. Then the viable bacteria were resuspended in PBS at 2 × 10^9^ CFU/ml. Each mouse was intraperitoneally injected with 0.5 ml bacterial suspension. Metformin (dissolved in PBS) or vehicle (PBS) was given intragastrically 1 h later at a dose of 250 mg/kg body weight for once. The mice were given *ad libitum* access to drinking water or water containing 1.5 mg/ml metformin. Mouse survival was monitored every 6 h for 4 days.

### Histopathology

Mice were sacrificed 8 h after bacterial infection. The liver was harvested, fixed in 4% neutral formaldehyde. Paraffin slice of the liver was stained with hematoxylin and eosin. Images were captured under the Zeiss Axio Observer D1 microscope armed with a color CCD (ZEISS).

### Statistical Analysis

All experiments were performed independently at least three times unless otherwise stated, with one representative experiment shown. Data are presented as mean ± SD. Statistical analysis was performed using GraphPad Prism 4.0 (GraphPad; San Diego, CA, USA). One-way analysis of variance followed by Tukey’s multiple comparison test was used to analyze the statistical significance among multiple groups and unpaired Student’s *t*-test between two groups. Kaplan–Meier survival curves were adopted for analysis of mice survival, and the statistical difference between two groups was determined using the log-rank (Mantel–Cox) test. *P* values < 0.05 were considered statistically significant.

## Results

### AMPK Agonist Metformin Enhances ATP-Induced Pyroptosis in Macrophages

**Figure 1:**
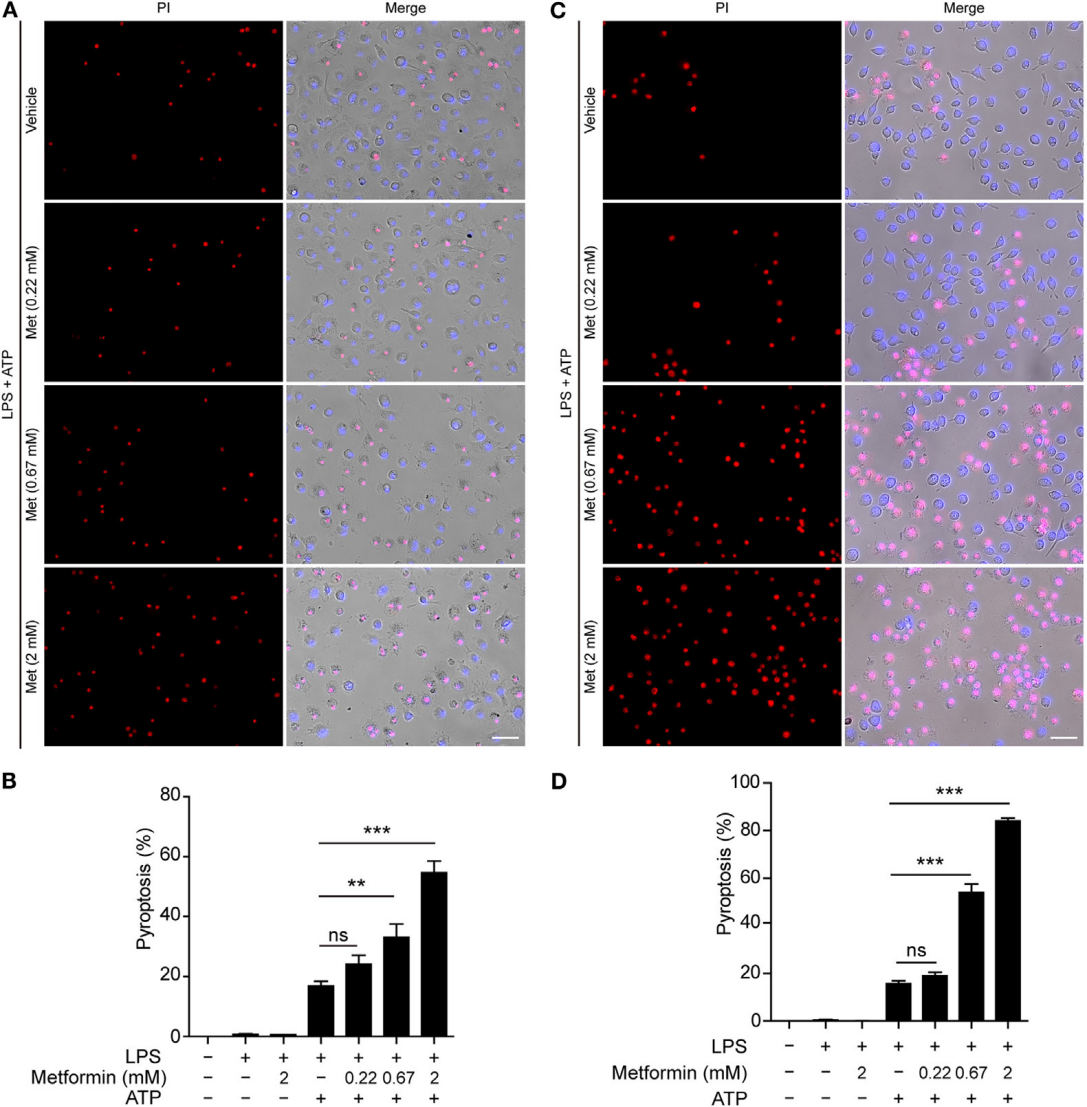
**Metformin enhanced adenosine triphosphate (ATP)-induced pyroptosis in lipopolysaccharide (LPS)-activated macrophages**. Mouse bone marrow-derived macrophages **(A,B)** and J774A.1 cells **(C,D)** were first primed with LPS (500 ng/ml) for 4 h, followed by treatment with indicated concentrations of metformin for 1 h. Subsequently, 2 mM ATP **(A)** or 3 mM ATP **(C)** was added to the medium for 30 min. Cells were stained by Hoechst 33342 (blue; for all cells) and propidium iodide (red; for pyroptotic cells) and images were captured using fluorescence microscopy, merged with bright-field images. Five independent experiments were performed (each in triplicates), and representative data from one experiment are shown. **(B,D)** Pyroptosis of five random fields each containing about 100 cells was calculated. Data are shown as mean ± SD (*n* = 5). Scale bars, 50 µm. Met, metformin. **P < 0.01; ***P < 0.001; and ns, not significant.

Although many studies have focused on how inflammasomes are assembled and activated, little is known about the regulatory signaling of NLRP3 inflammasome activation upon extracellular ATP stimulation. As dramatic metabolic changes occur during macrophage activation (22), it is of great interest whether AMPK, a key regulator of energy metabolism, regulates ATP-induced inflammasome activation in LPS-primed macrophages. To explore this problem, LPS-activated BMDMs were treated with serial concentrations of metformin, a well-known AMPK agonist, followed by ATP treatment. First, we observed the pyroptotic cells (PI permeable) and calculated their ratio to the total cells (indicated by nuclear dye Hoechst 33342). As expected, ATP induced pyroptosis in the BMDMs; and metformin treatment further increased the pyroptosis induced by LPS and ATP treatment (Figures 1A,B). However, metformin alone did not induce cell death in both control and LPS-treated cells without ATP treatment (Figure S1A in Supplementary Material). Similar results were observed in mouse macrophage J774A.1 cell line (Figures 1C,D; Figure S1B in Supplementary Material). Considering that metformin is an AMPK agonist, these results suggested that AMPK signaling might have contributed to ATP-induced pyroptosis in LPS-activated macrophages.

### AMPK Signaling Is Suppressed by LPS but Reactivated by Extracellular ATP

**Figure 2:**
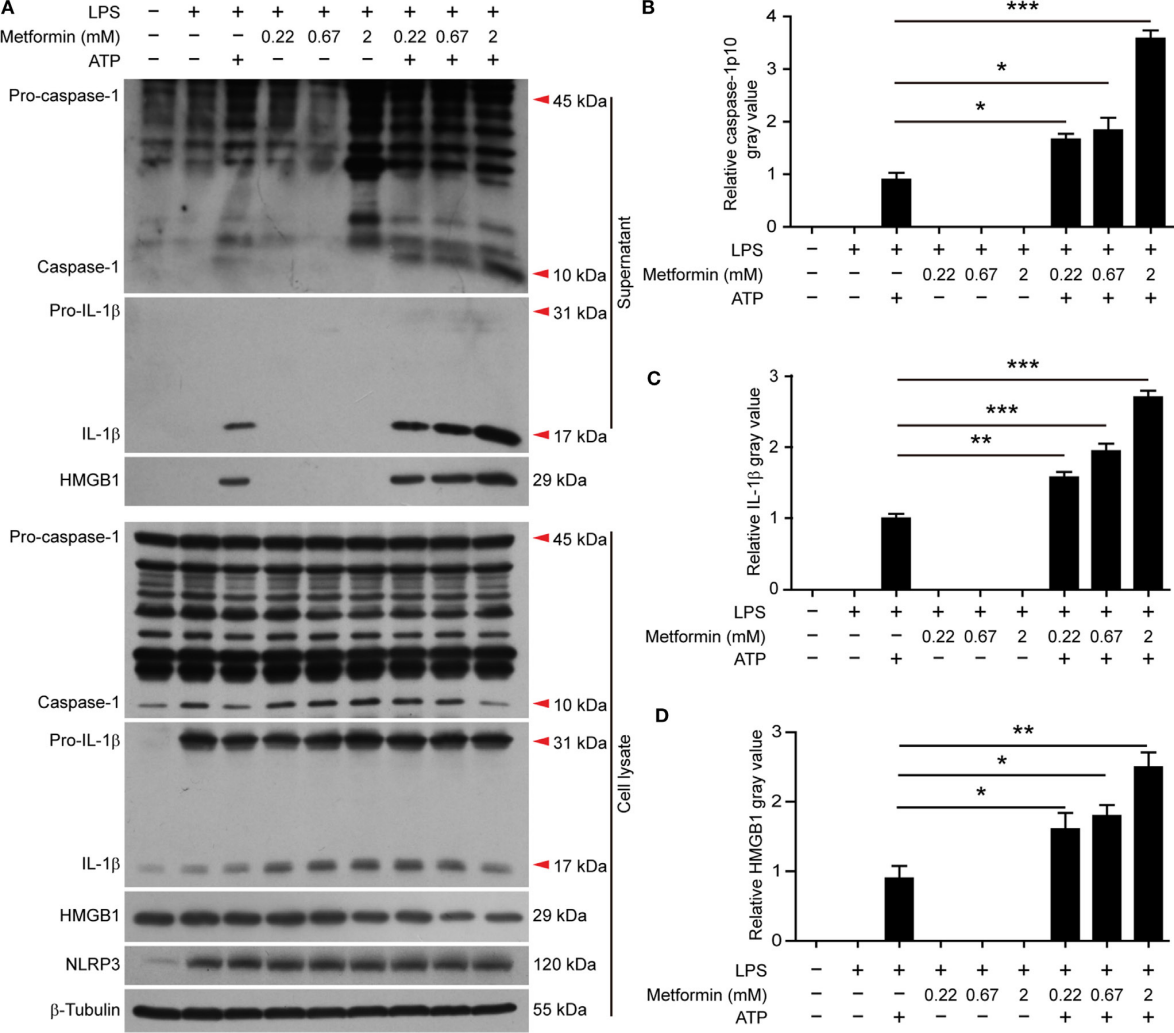
**Metformin increased adenosine triphosphate-induced inflammasome activation in bone marrow-derived macrophages**. **(A)** Cells were treated as described in Figure 1. Indicated protein levels in both cell lysates and supernatants were evaluated by western blotting. β-Tubulin was used as a loading control for cell lysates. **(B–D)** The quantitative analyses of the active caspase-1p10 **(B)**, mature interleukin-1β **(C)**, and HMGB1 **(D)** levels in the supernatants **(A)** are shown. Data are shown as mean ± SD (*n* = 3). One representative experiment from three independently performed experiments with similar results is shown. *P < 0.05; **P < 0.01; and ***P < 0.001.

It has been observed that the activity of AMPK can be dampened in macrophages by LPS, free fatty acid, or other stimulators (28). But recent studies showed that AMPK signaling is dramatically increased in LPS-activated macrophages after ATP treatment (21, 22), accompanied by pyroptosis and mature IL-1β release (22). Consistent with these studies, western blot analysis demonstrated that LPS (alone) treatment led to a marked increase of NLRP3 and pro-IL-1β protein expression in BMDMs, but it did not induce the release of active caspase-1p10, mature IL-1β, and HMGB1 (Figures 2A–D, supernatants). Upon ATP triggering, however, active caspase-1p10 fragment, HMGB1, and mature IL-1β (17 kDa) were released into the supernatants (Figures 2A–D). Moreover, the release of these contents from the cells in response to ATP stimulation can be further promoted by metformin treatment (Figures 2A–D). As the release of mature IL-1β is dependent on pyroptosis (4, 10), our results suggested that the augmentation of AMPK signaling by metformin enhanced LPS + ATP-induced inflammasome activation and pyroptosis.

**Figure 3:**
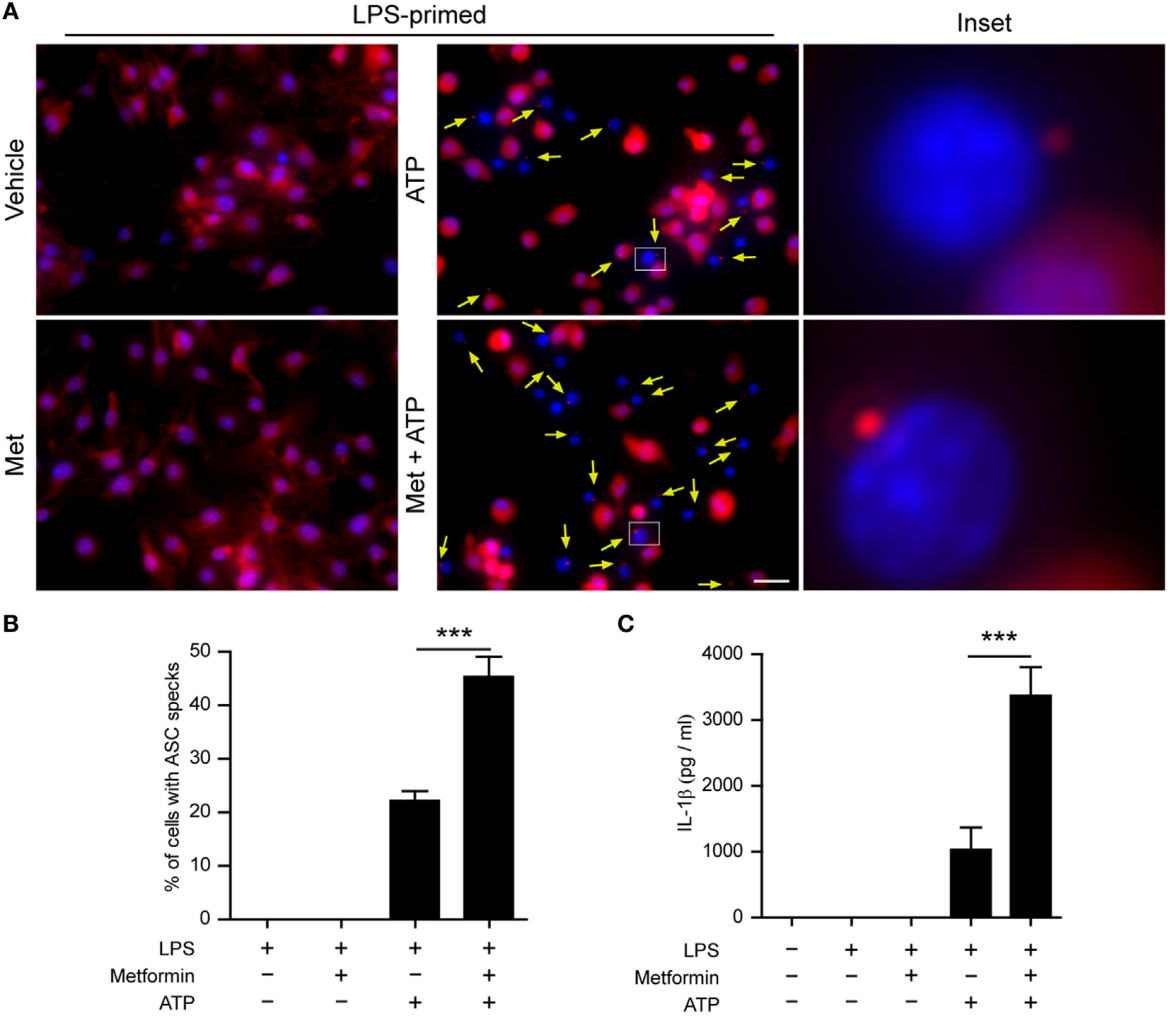
**Metformin treatment increased adenosine triphosphate (ATP)-induced ASC speck formation and interleukin (IL)-1β secretion in bone marrow-derived macrophages (BMDMs)**. **(A)** Mouse BMDMs were seeded in glass-bottomed dishes and primed with lipopolysaccharide (500 ng/ml, in complete DMEM medium) for 4 h, and then treated with metformin (2 mM, in Opti-MEM) for 1 h. Subsequently, 2 mM ATP was added to the medium for 30 min. The distribution of ASC (red) was revealed by immunofluorescence microscopy with the nuclei being stained with Hoechst 33342 (blue). The images for ASC and nuclei were captured, respectively, and merged together. The experiment was performed twice, with one representative set of images shown. The yellow arrows indicate ASC specks. An enlarged inset for each image with an ASC speck is also shown. Scale bar, 20 µm; Met, metformin. **(B)** The ratios of cells containing ASC specks were calculated by the number of cells with ASC specks relative to the total number of cells from five random fields each containing about 50 cells. Data are shown as mean ± SD (*n* = 5). **(C)** Quantification of soluble IL-1β levels in the cell culture supernatants by cytometric bead array assay. Data are shown as mean ± SD (*n* = 3). ***P < 0.001.

Next, we performed two additional assays to corroborate the augmentation effect of metformin on ATP-induced inflammasome activation. First, the subcellular distribution of ASC in BMDMs was observed by immunofluorescence microscopy. ASC is an adaptor protein that mediates the recruitment of caspase-1 to the NLRP3 inflammasome to form a large speck in the cell, and thus activates caspase-1 (29). The results showed that ASC was evenly distributed in LPS and vehicle-treated BMDMs (Figure 3A). After ATP treatment, ASC specks could be observed in ~20% of LPS-primed cells (Figures 3A,B), indicating the activation of inflammasomes as having been indicated previously (30). Furthermore, metformin treatment increased the ratio of cells containing ASC specks induced by ATP to >40% (Figures 3A,B). Second, the CBA assay was used to detect soluble IL-1β in the supernatants. The results showed that LPS + ATP treatment induced a significant release of IL-1β from BMDMs, which could be greatly promoted by metformin treatment (Figure 3C).

**Figure 4:**
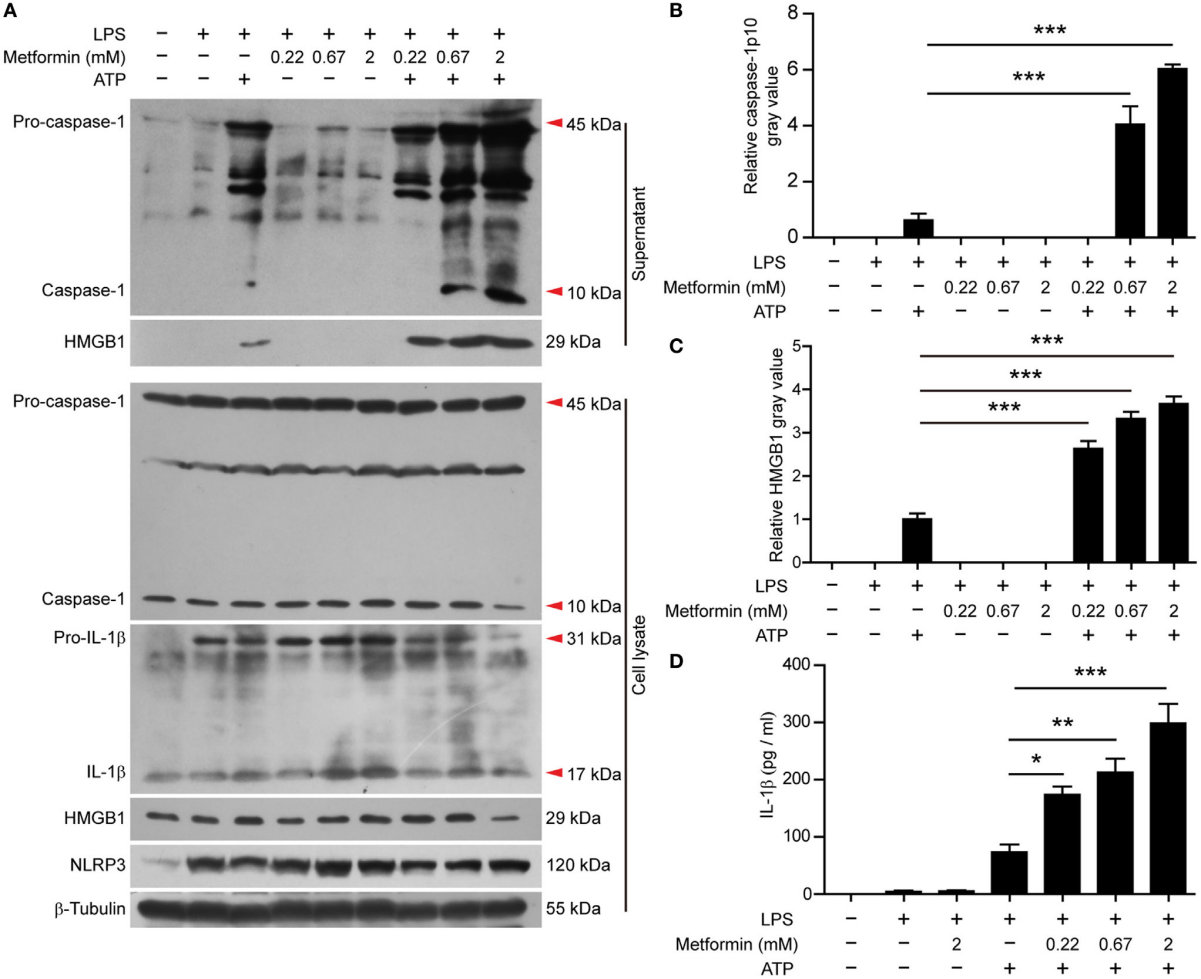
**Metformin enhanced adenosine triphosphate-induced inflammasome activation in J774A.1 cells**. **(A)** Cells were treated as described in Figure 1. Indicated protein levels in both cell lysates and supernatants were evaluated by western blotting. β-Tubulin was used as a loading control for cell lysates. **(B,C)** Quantitative analyses of active caspase-1p10 **(B)** and HMGB1 **(C)** levels in the supernatants **(A)**. **(D)** Quantification of soluble interleukin-1β levels in the cell culture supernatants by cytometric bead array assay. Data are shown as mean ± SD (*n* = 3). One representative experiment from three independently performed experiments with similar results is shown. *P < 0.05; **P < 0.01; and ***P < 0.001.

In J774A.1 cells, we also observed that LPS induced the expression of NLRP3 and pro-IL-1β (Figure 4A, cell lysates). Upon ATP treatment, both active caspase-1p10 and HMGB1 could be released into the supernatants, and metformin dose-dependently increased their release (Figures 4A–C). Although soluble IL-1β was hardly detectable in the precipitated proteins of the culture supernatants by western blotting, CBA assays could detect such low levels of IL-1β and the result showed that LPS + ATP induced the release of IL-1β and metformin dose-dependently enhanced its release (Figure 4D).

It seemed that metformin did not influence the constitutive expression of pro-caspase-1 (45 kDa) and the induced-expression of pro-IL-1β (31 kDa) and NLRP3 (120 kDa) by LPS in the two cell types, nor did it induce the release of mature IL-1β, active caspase-1p10, and HMGB1 as well as the formation of ASC specks in the absence of ATP treatment (Figures 2–4).

**Figure 5:**
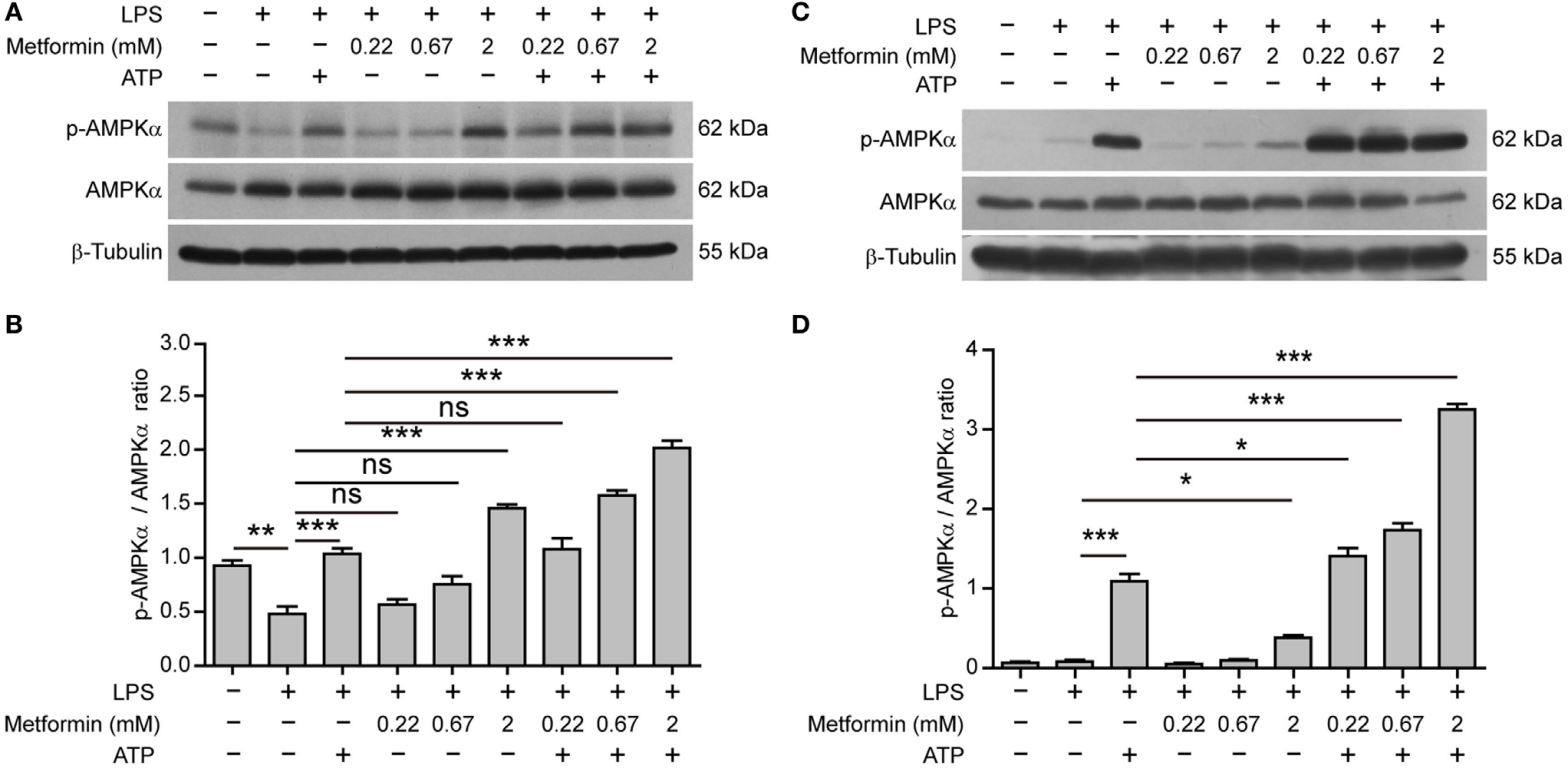
**Metformin enhanced adenosine triphosphate-induced AMP-activated protein kinase (AMPK) signaling in bone marrow-derived macrophages [BMDMs, (A,B)] and J774A.1 cells (C,D)**. **(A,C)** BMDMs and J774A.1 cells were treated as described in Figure 1, respectively. Indicated protein levels of p-AMPKα and AMPKα were evaluated by western blotting. β-Tubulin was used as a loading control for cell lysates. **(B,D)** Quantification of p-AMPKα levels relative to AMPKα in BMDMs **(B)** and J774A.1 cells **(D)**. Data are shown as mean ± SD (*n* = 3). One representative experiment from three independently performed experiments with similar results is shown. *P < 0.05; **P < 0.01; ***P < 0.001; and ns, not significant.

Interestingly, in BMDMs, AMPK activity (reflected by its phosphorylation at Thr172) was suppressed by LPS alone (Figures 5A,B); while in J774A.1 cells, AMPK activity was low in both control and LPS (alone) groups (Figures 5C,D). Upon ATP treatment, however, AMPK was reactivated in both cell types. Furthermore, metformin treatment dose-dependently increased the AMPK activation as compared to their respective control (LPS alone or LPS + ATP) (Figures 5A–D).

Altogether, these results suggested that elevated AMPK signaling was associated with inflammasome activation and pyroptosis in LPS-primed macrophages upon ATP stimulation.

### Suppression of AMPK Signaling Attenuates Pyroptosis in Macrophages

**Figure 6:**
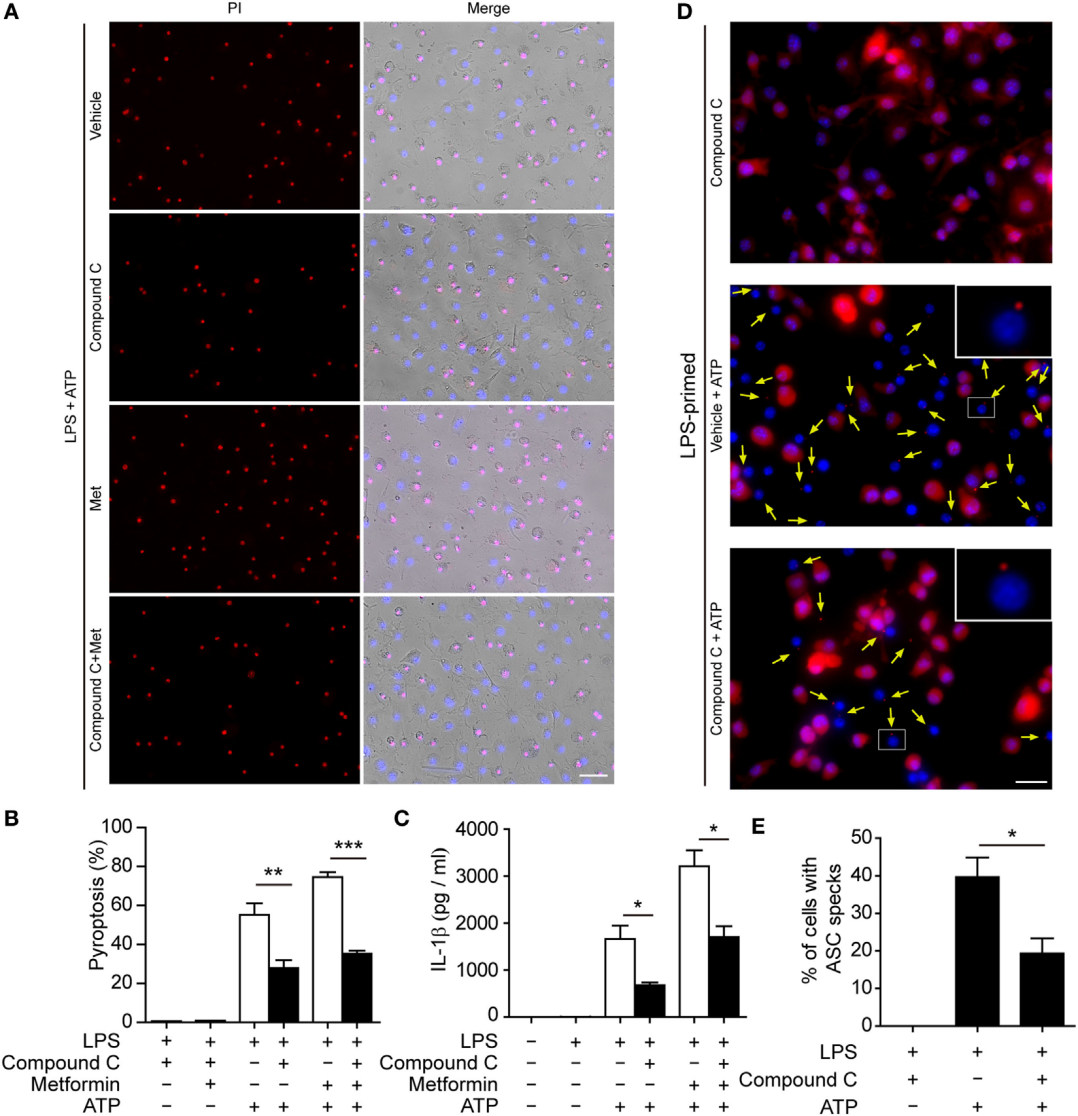
**Pharmacological blockade of AMP-activated protein kinase signaling suppressed adenosine triphosphate (ATP)− or ATP+ metformin-induced pyroptosis in mouse bone marrow-derived macrophages (BMDMs)**. BMDMs were first primed with lipopolysaccharide (500 ng/ml) for 4 h, and then pretreated with compound C (20 µM in Opti-MEM) for 1 h and metformin (2 mM in Opti-MEM) for 1 h. Subsequently, 2 mM ATP was added to the medium for 30 min. **(A)** Pyroptotic cells were revealed by their fluorescence of propidium iodide (PI) (red) staining. The images of PI and Hoechst 33342 (blue; for all cells) staining were merged with bright-field images. Scale bar, 50 µm; Met, metformin. **(B)** Pyroptosis ratios were calculated by the number of PI-positive cells relative to the total number of cells in five random fields each containing about 100 cells. Data are shown as mean ± SD (*n* = 5). **(C)** The levels of soluble interleukin-1β in the cell culture supernatants were evaluated by cytometric bead array assay. Data are shown as mean ± SD (*n* = 3). One representative experiment from three independently performed experiments with similar results is shown. **(D)** The subcellular distribution of ASC (red) was revealed by immunofluorescence microscopy with the nuclei being stained with Hoechst 33342 (blue). Scale bar, 20 µm. **(E)** The ratios of cells containing ASC specks were calculated by the number of cells with ASC specks relative to the total number of cells from five random fields (each containing about 50 cells). Data are shown as mean ± SD (*n* = 5). One experiment of two independently performed experiments with similar results is shown. *P < 0.05; **P < 0.01; and ***P < 0.001.

**Figure 7:**
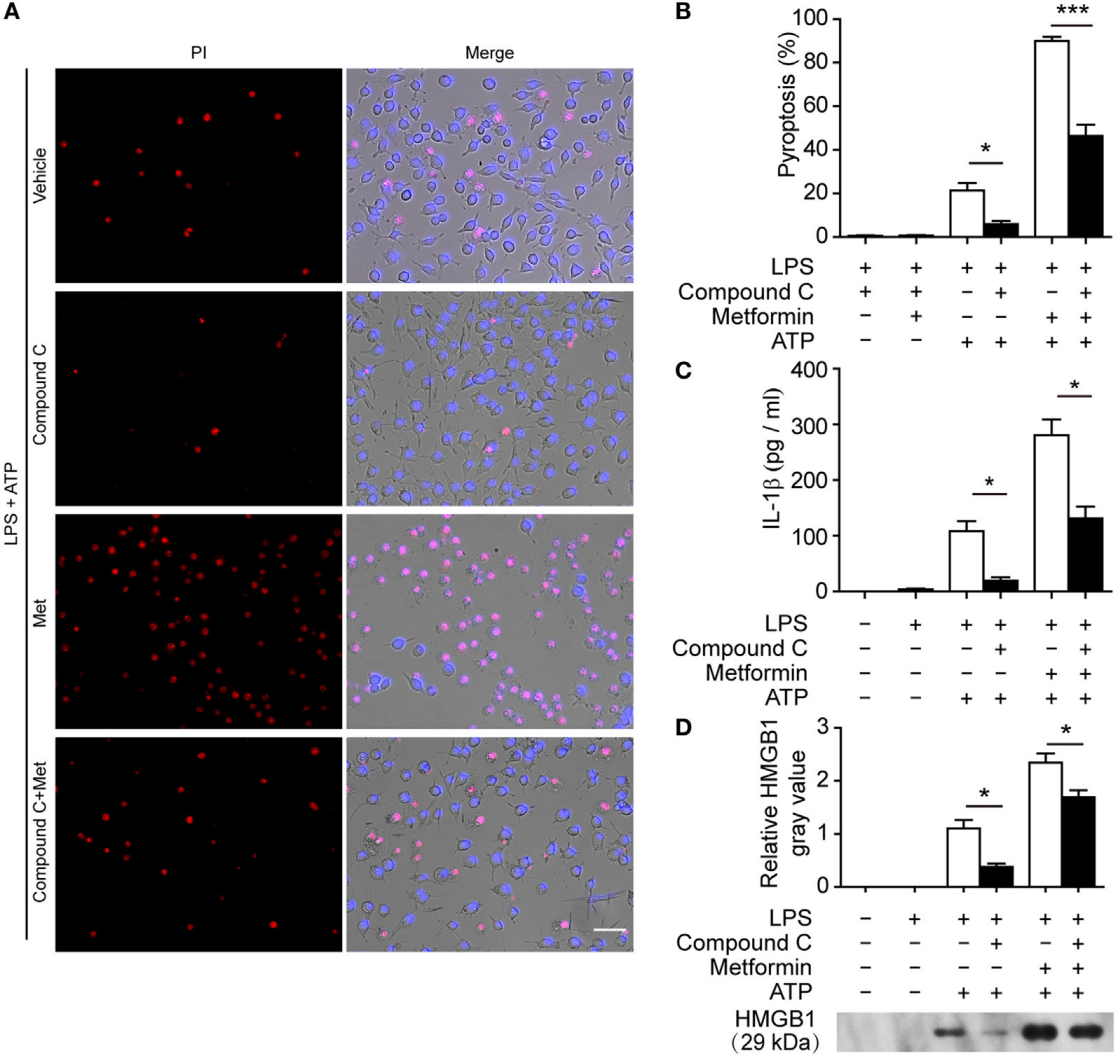
**Pharmacological blockade of AMP-activated protein kinase signaling suppressed adenosine triphosphate (ATP)− or ATP+ metformin-induced pyroptosis in J774A.1 cells**. J774A.1 cells were first primed with lipopolysaccharide (LPS) (500 ng/ml) for 4 h, and then pretreated with compound C (20 µM in Opti-MEM) for 1 h and metformin (2 mM in Opti-MEM) for 1 h. Subsequently, 3 mM ATP was added to the medium for 30 min. **(A)** Pyroptotic cells were revealed by their fluorescence of propidium iodide (PI) (red) staining and the nuclei were stained with Hoechst 33342 (blue, for all cells). The images of PI and Hoechst 33342 staining were merged with bright-field images. Scale bar, 50 µm; Met, metformin. **(B)** Pyroptosis ratios were calculated by the number of PI-positive cells relative to the total number of cells in five random fields each containing about 100 cells. Data are shown as mean ± SD (*n* = 5). **(C)** The levels of soluble interleukin-1β in the supernatants were evaluated by cytometric bead array assay (*n* = 3). **(D)** Soluble HMGB1 in the culture supernatants were precipitated and evaluated by western blotting, setting the blot gray value of LPS + ATP group as 1.0 (*n* = 3). *P < 0.05 and ***P < 0.001.

To further explore whether AMPK activation contributes to ATP-induced pyroptosis in LPS-activated macrophages, BMDMs were first primed with LPS and then treated with compound C, a well-known pharmacological AMPK inhibitor (31, 32). Subsequently, the cells were treated with ATP. As shown in Figures 6A,B, compound C significantly suppressed ATP-induced pyroptosis in LPS-primed BMDMs. Supporting this, compound C also inhibited the release of IL-1β (Figure 6C) and the formation of ASC specks (Figures 6D,E) in these cells. Similar results were observed in J774A.1 cells. Compound C not only suppressed ATP-induced pyroptosis (Figures 7A,B) but also inhibited the secretion of IL-1β (Figure 7C) and HMGB1 (Figure 7D) from J774A.1 cells. Moreover, compound C significantly attenuated the effect of metformin on promoting ATP-induced pyroptosis (Figures 6A,B and 7A,B), as well as the release of IL-1β (Figures 6C and 7C) and HMGB1 (Figure 7D) in LPS-primed BMDMs and J774A.1 cells, respectively. Without ATP treatment, compound C or compound C plus metformin did not induce pyroptosis in these two macrophage types (Figures S1C,D in Supplementary Material).

**Figure 8:**
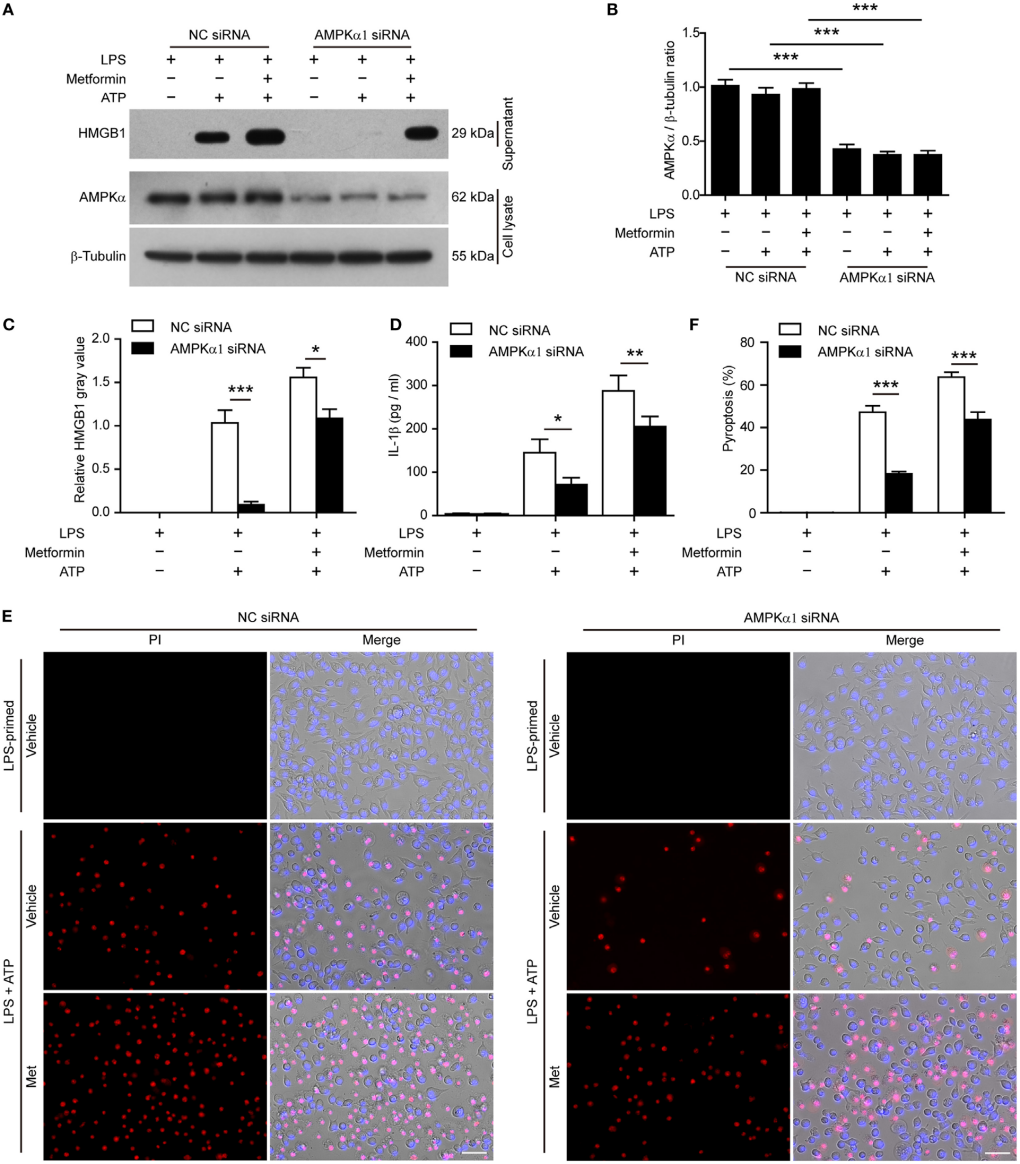
**Knockdown of *AMPK*α*1* expression attenuated adenosine triphosphate (ATP)-induced pyroptosis**. The expression of AMPKα1 in J774A.1 cells were knocked down by small-interfering RNA (siRNA) targeting *AMPK*α*1* gene. Negative control (NC) siRNA was recruited as a knockdown control. Seventy-two hours after knockdown, the cells were primed with lipopolysaccharide (LPS) (500 ng/ml) for 4 h, followed by incubation with metformin (2 mM) for 1 h prior to ATP (3 mM) stimulation for 30 min. **(A)** The expression of AMPKα after knockdown was detected by western blotting. β-Tubulin was used as loading control. **(B)** Quantitative analysis of AMPKα levels relative to that of β-tubulin in western blotting **(A)**. **(C)** The soluble HMGB1 levels were precipitated and evaluated by western blotting **(A)**, setting the blot gray value of LPS + ATP group as 1.0. **(D)** The soluble interleukin-1β in the supernatants of each treatment group was evaluated by cytometric bead array assay (*n* = 3). *P < 0.05; **P < 0.01; and ***P < 0.001. **(E)** Pyroptotic cells were revealed by their fluorescence of propidium iodide (PI) (red) staining in conjunction with Hoechst 33342 (blue) staining (for all cells). The images of PI and Hoechst 33342 fluorescence were merged with bright-field images. Met, metformin. **(F)** Pyroptosis ratios were calculated by the number of PI-positive cells relative to total number of cells in five random fields each containing about 100 cells. Data are shown as mean ± SD (*n* = 5). Scale bars, 50 µm. ***P < 0.001.

To further confirm the pharmacological data, we performed further experiments in J774A.1 cells in which the expression of AMPKα had been genetically knocked down by *AMPK*α*1*-specific siRNA. When AMPKα expression was nearly reduced by 65% after siRNA knockdown (Figures 8A,B), the release of HMGB1 (Figures 8A,C, western blotting) and IL-1β (Figure 8D, CBA assay) by ATP or metformin plus ATP treatment was significantly reduced as compared to that of NC siRNA treated cells. Consistent with the reduction of these inflammasome activation markers, ATP-induced pyroptosis was also significantly decreased (Figures 8E,F). Notably, metformin-mediated augmentation of pyroptosis by ATP stimulation was also markedly attenuated by *AMPK*α knockdown (Figures 8E,F). Taken together, these results indicated that AMPK signaling had contributed to ATP-induced inflammasome activation and pyroptosis in LPS-primed murine macrophages.

### Metformin Administration Aggravates Mouse Bacterial Sepsis

**Figure 9:**
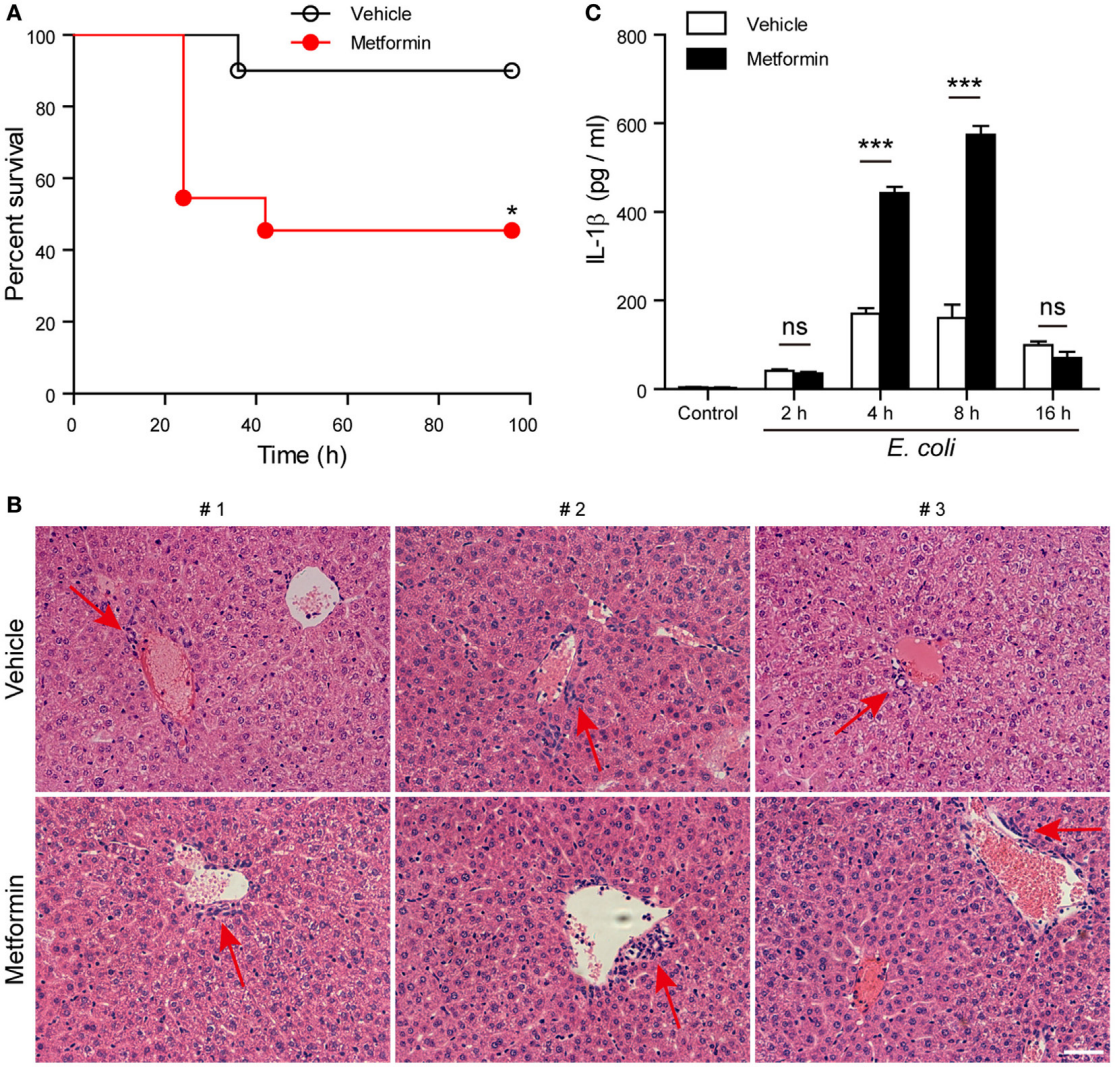
**Metformin administration aggravated bacterial sepsis in mice**. C57BL/6 mice were intraperitoneally injected with viable *Escherichia coli* at a dose of 1 × 10^9^ CFU/mouse. After 1 h, the mice were intragastrically given with metformin at a dose of 250 mg/kg body weight or vehicle. **(A)** Kaplan–Meier survival curves of the mice monitored every 6 h for 4 days. Each group contained 10 mice and three independent experiments were performed with one set of data presented. *P < 0.05. **(B)** Histopathological analysis of liver sections after bacterial infection. Red arrows indicated the infiltrated inflammatory cells. **(C)** Serum interleukin-1β levels after bacterial infection. Data are shown as mean ± SD (*n* = 5). Each group contained five mice and two independent experiments were performed with one set of data presented. Scale bar, 50 µm. ***P < 0.001 and ns, not significant.

**Figure 10:**
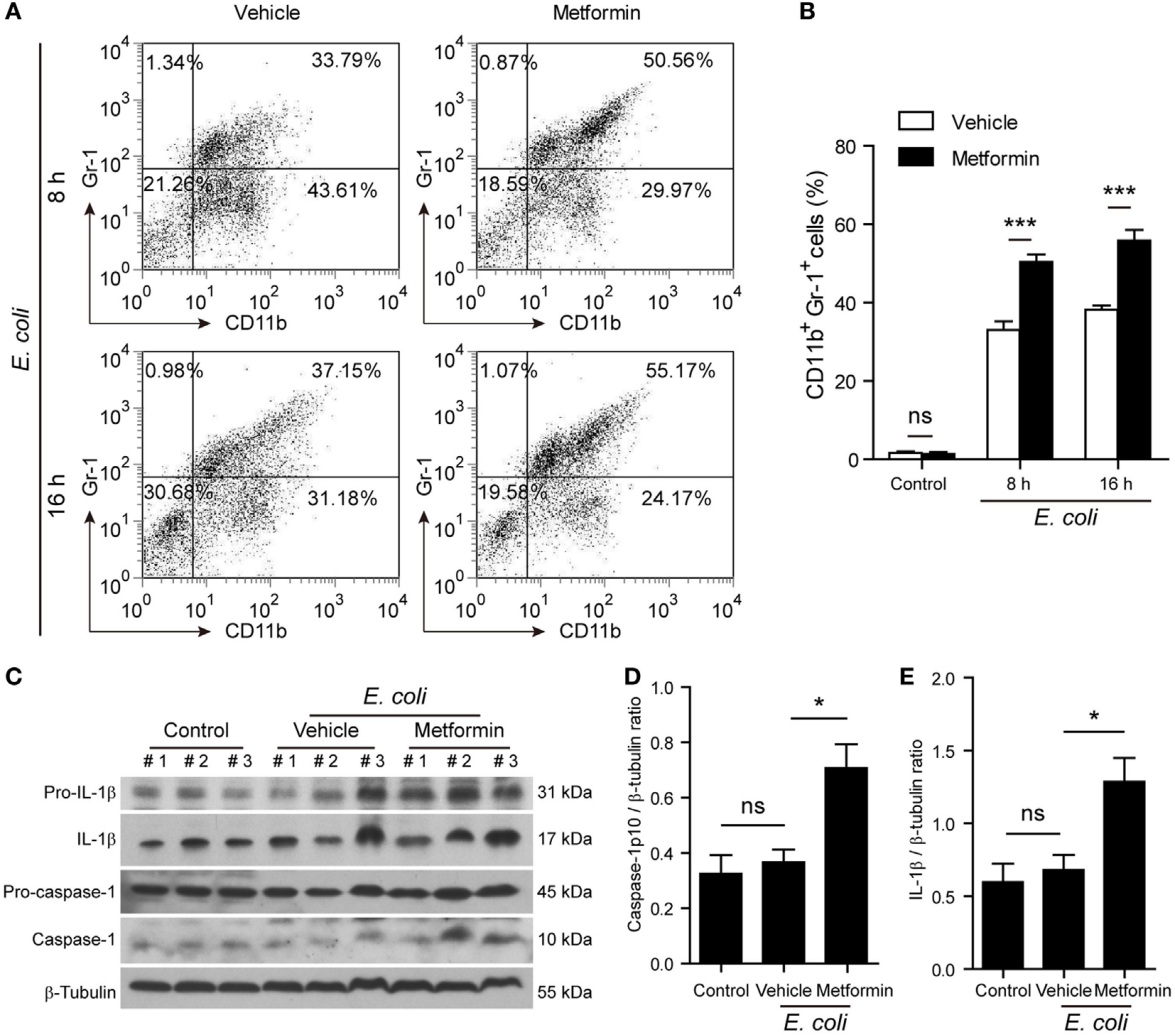
**Metformin administration exacerbated inflammatory responses in bacterial sepsis**. C57BL/6 mice were treated as described in Figure 9. **(A,B)** Flow cytometric analysis of neutrophils (CD11b^+^Gr-1^+^) in the peritoneal cavity after bacterial infection. Representative dot-plots are shown in **(A)** and histograms are shown in **(B)** as mean ± SD (*n* = 5). ***P < 0.001. **(C–E)** Western blot analysis of protein expression in the liver 8 h after the mice being infected with bacteria. The blots of three individual mice in each group were displayed **(C)**. The relative protein levels of active caspase-1p10 **(D)** and mature interleukin-1β **(E)** were quantified by densitometry. Data are shown as mean ± SD (*n* = 5). *P < 0.05 and ns, not significant.

Finally, we investigated whether metformin administration enhanced systemic inflammation *in vivo* using a mouse model of bacterial sepsis. Mice were first intraperitoneally injected with a sublethal dose of live *E. coli* according to previous studies (9, 27). The bacterial infection at a dose of 1 × 10^9^ CFU/mouse caused only ~10% mortality in the mice treated with vehicle after 24 h, but metformin administration accelerated the onset of death (within 24 h) and significantly increased the mortality of the mice to ~50% during the period of 4-day observation (Figure 9A), suggesting that metformin exacerbated the severity of bacterial sepsis. Consistent with this result, histopathological analysis showed that bacterial infection led to infiltration of inflammatory cells in the liver in both metformin and vehicle groups, but the mice in the metformin group showed more inflammatory cell infiltration and tissue injury around their hepatic vascular walls (Figure 9B). In further support of this, metformin-treated mice had a marked increase in their serum levels of IL-1β at 4-h and 8-h time points when compared with controls (vehicle treated) (Figure 9C), indicating increased inflammasome activation in metformin-treated mice upon bacterial infection. In addition, the percentages of neutrophils (CD11b^+^Gr-1^+^ cells) in the peritoneal cavity were greatly increased in metformin-treated mice in comparison to vehicle (Figures 10A,B). As indicators of inflammasome activation and pyroptosis (10), hepatic mature IL-1β and active caspase-1p10 protein levels were higher in the metformin group as compared to control and vehicle group, as revealed by western blotting (Figures 10C–E). These results suggested that bacterial infection had systemically induced inflammasome activation and IL-1β maturation, and that metformin treatment enhanced such inflammasome activation and organ injury, culminating in aggravated bacterial sepsis in mice.

## Discussion

During bacterial infection or tissue damage, ATP is released from the intracellular compartments of both the host and bacterial cells into the extracellular milieu (9, 33, 34). The extracellular ATP, acting as a second signal for canonical NLRP3 inflammasome activation, induces pyroptosis in innate immune cells including macrophages (6). However, the signaling pathways regulating the pyroptosis are largely unknown, although some of which have been uncovered recently (22, 35). In this study, we provided several lines of evidence to reveal that AMPK signaling regulated ATP-induced inflammasome activation and pyroptosis in LPS-primed murine macrophages. First, ATP treatment has been shown to activate AMPK in LPS-primed macrophages by previous studies (21, 22) and ours. Augmentation of AMPK signaling by its agonist (metformin) robustly enhanced ATP-induced pyroptosis. Second, pharmacological blockade of AMPK activity by its inhibitor (compound C) not only suppressed ATP-induced pyroptosis, but also mitigated the augmentation effect of metformin on the pyroptosis. Third, genetic knockdown of AMPKα, the kinase subunit of AMPK, also attenuated ATP-induced pyroptosis either with or without metformin treatment. Finally, *in vivo* administration of metformin increased the systemic levels of IL-1β and hepatic inflammation in bacterial infected mice. Therefore, AMPK signaling had been involved in regulating ATP-induced inflammasome activation and pyroptosis.

One interesting phenomenon is that AMPK is suppressed in macrophages when stimulated with LPS or other inflammatory stresses (28), but it is sharply switched to an activated state when the cells are exposed to a second danger signal (e.g., ATP) (22). The cause for this sharp switch is still unknown. AMPK is a key regulator of cellular energy metabolism (36). Under nutritional and other cellular stresses, AMPK is activated and thus inhibits the mechanistic target of rapamycin complex 1 (mTORC1) activity *via* phosphorylation of Raptor (a component of mTORC1) or TSC2 (a suppressor of mTORC1) leading to the down-regulation of protein synthesis (37, 38). It also phosphorylates and thus inactivates acetyl-CoA carboxylase (39), which catalyzes the carboxylation of acetyl-CoA to provide malonyl-CoA for the biosynthesis of fatty acids (40). Hence, the biosynthesis of cellular components including proteins and lipids is suppressed upon AMPK activation (41). As a key regulator of energy metabolism, AMPK is likely involved in regulating such remarkable energy changes during the process of pyroptosis. Indeed, we found in this study that the AMPK signaling could regulate ATP-induced inflammasome activation and pyroptosis.

Pyroptosis is one critical consequence of ATP-induced inflammasome activation. During this process, several kinds of membranous channels or pores can be opened or formed in the cell membrane, allowing the release of intracellular contents (11). Mechanistically, ATP induces inflammasome activation mainly through its action on cell membrane receptor P2X_7_R (42, 43). After ATP engagement, P2X_7_R molecules form a non-selective cation channel for efflux of K^+^ ion. If P2X_7_R is persistently activated, it may further recruit pannexin-1 to form pores that allow IL-1β release (44–46) and induction of cell death. Importantly, ATP-induced K^+^ efflux can trigger NLRP3 inflammasome assembly, leading to caspase-1 activation. The latter consequently cleaves gasdermin D to produce its N-terminal fragment, which forms another type of membrane pores to induce pyroptosis (47–49). The resultant pores have an inner diameter around 32 nm (48), thus allowing the export of mature IL-1β, HMGB1, and other cellular components (50). It is hypothesized that the channel opening or pore-forming induced by extracellular ATP is regulated by AMPK signaling. Supporting this, ATP-triggered P2X_7_R can integrate PI3K/AKT and AMPK-mTOR signaling pathways to induce cell death in tumor cells (51). In line with this, our preliminary observation also revealed a decrease in the mTORC1 activity during the process of ATP-induced pyroptosis (data not shown), concomitant with the AMPK activation. In addition, AMPK signaling has been reported to trigger a rapid redistribution of intracellular GLUT4 onto the cell membrane and thus facilitate glucose uptake (52, 53). However, our preliminary experiments showed that the distribution of P2X_7_R on the cell membrane upon ATP stimulation was not enhanced by AMPK agonist metformin treatment, at least in BMDMs (data not shown), suggesting additional mechanisms may be involved in this process.

Beyond P2X_7_R, NLRP3 is a critical component mediating the activation of caspase-1 and thus pyroptosis upon ATP treatment. The activity of NLRP3 is regulated by phosphorylation. Once dephosphorylated at tyrosine 861 by tyrosine phosphatase non-receptor 22 (PTPN22), NLRP3 is activated to allow caspase-1 activation and IL-1β release (35). It has also been revealed that mTORC1 signaling could regulate NLRP3 inflammasome activation in macrophages by upregulation of hexokinase-1-dependent glycolysis (22). As we found no significant change in the NLRP3 protein levels upon AMPK activation by metformin, it is likely that AMPK regulates the phosphorylation of NLRP3 directly or indirectly through other kinases or phosphatases like PTPN22. More research is thus warranted to reveal the precise mechanism of AMPK signaling in regulating NLRP3 inflammasome activation and pyroptosis.

Metformin is a first-line drug for type 2 diabetes (T2D). As an AMPK agonist (32), metformin has been demonstrated to suppress both hepatic gluconeogenesis and lipid genesis (54, 55) in patients with obesity and T2D or animal models. Nonetheless, the exact mechanism underlying the action of metformin on obesity and diabetes is still not completely understood. NLRP3 inflammasome activation is upregulated in T2D patients (56) and a previous work has demonstrated that NLRP3 expression in adipose tissues impairs insulin-sensitivity (13). However, spontaneous pyroptosis among these activated cells may be limited. Instead, the AMPK activity is inhibited in patients with obesity and T2D and their tissue-resident macrophages are activated (57). These macrophages continuously release inflammatory cytokines to contribute to and maintain the chronic inflammatory diseases (58–60). Based on these findings and ours, we propose a possibility that metformin may provide beneficial effect to T2D patients by promoting pyroptosis to eliminate the chronically activated macrophages, particularly those in adipose tissues, thus finally alleviating the chronic inflammation in T2D patients.

Our data appeared inconsistent with some previous studies, which showed that metformin treatment suppressed intracellular levels of pro-IL-1β without influencing IL-1β secretion (61) or reduced IL-1β and IL-18 secretion (56). This discrepancy between the results of those previous studies and ours may be due to different experimental settings (61) or recruitment of different cellular models (56). Although previous reports have shown that another AMPK agonist AICAR (at a low dose of 100 µM) does not influence LPS + ATP-induced IL-1β secretion (22, 59), higher doses of AICAR may show different effects. Indeed, we found that a high dose of AICAR (2 mM, a commonly used concentration to activate AMPK) had similar effects as metformin did on ATP-induced pyroptosis in J774A.1 cells (data not shown).

In summary, our study revealed a link between AMPK signaling and ATP-induced pyroptosis in LPS-activated macrophages. Enhancing AMPK activity could significantly augment ATP-induced inflammasome activation and pyroptosis in macrophages, leading to increased release of IL-1β and HMGB1. Our finding may be of significance on investigating new AMPK-targeting therapies for diseases involving inflammasome activation, including obesity, diabetes, and bacterial sepsis.

## Ethics Statement

All animal experiments were performed according to the guidelines for the care and use of animals approved by the Committee on the Ethics of Animal Experiments of Jinan University.

## Author Contributions

Q-BZ, H-XW, and L-HX performed *in vitro* studies; C-GL, Y-DL, and W-JB conducted animal studies; H-XW, C-GL, and HP analyzed the data; D-YO and X-HH supervised the study; D-YO, X-HH, and H-XW wrote the paper.

## Conflict of Interest Statement

The authors declare that the research was conducted in the absence of any commercial or financial relationships that could be construed as a potential conflict of interest. The reviewer FS and handling Editor declared their shared affiliation, and the handling Editor states that the process nevertheless met the standards of a fair and objective review.
