# Supplementary material for: ATP-Induced Inflammasome Activation and Pyroptosis Is Regulated by AMP-Activated Protein Kinase in Macrophages
Source: Front Immunol. 2016 Dec 12;7:597. doi: 10.3389/fimmu.2016.00597 (PMC5149551; doi:10.3389/fimmu.2016.00597)
Supplement: Supplementary file 1 [file Presentation_1.PDF]

Supplementary figure

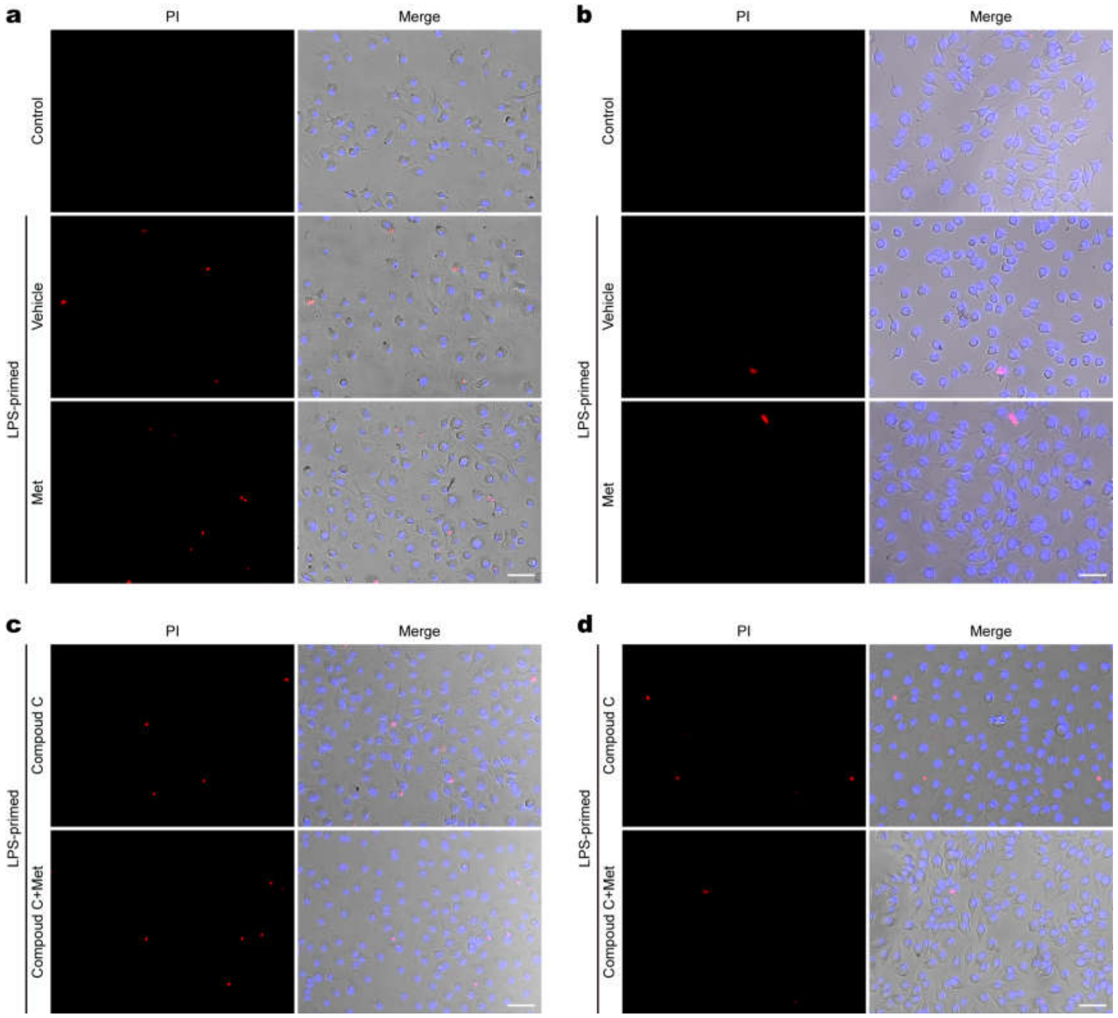

Supplementary Figure 1 | LPS or metformin *per se* did not induce pyroptosis in macrophages. (a, b) Mouse bone marrow-derived macrophages (BMDMs) **(a)** and J774A.1 cells **(b)** were primed with 500 ng/ml LPS for 4 h, followed by treatment with 2 mM metformin or vehicle (PBS) for 1 h. Finally, the cells were stained with 2 µg/ml propidium iodide (PI) and 5 µg/ml Hoechst 33342. Images of PI and Hoechest 33342 fluorescence were captured by a ZEISS microscope, merged with bright-field images. **(c, d)** Mouse BMDMs (c) and J774A.1 cells **(d)** were primed with 500 ng/ml LPS for 4 h, followed by sequential treatment with compound C for 1 h and metformin for 1 h, respectively. Finally, the cells were stained with PI and Hoechst 33342 and observed under the ZEISS microscope as described in **(a, b)**. Scale bars, 50 µm.
